# Supplementary material for: COZOID: contact zone identifier for visual analysis of protein-protein interactions
Source: BMC Bioinformatics. 2018 Apr 6;19:125. doi: 10.1186/s12859-018-2113-6 (PMC5889581; doi:10.1186/s12859-018-2113-6)
Supplement: Supplementary file 2 — Software build. Executable binary file of the software tool. (ZIP 118,784 kb) [file 12859_2018_2113_MOESM2_ESM.zip › cozoid/java/docs/beansbinding-1.2.1-doc.zip!/org/jdesktop/el/class-use/ELContext.html]

Uses of Class org.jdesktop.el.ELContext (Beans Binding)


---


|  |  |  |
| --- | --- | --- |
| **Overview** \| **Package** \| **Class** \| **Use** \| **Tree** \| **Deprecated** \| **Index** \| **Help** | |  |
| PREV   NEXT | **FRAMES**    **NO FRAMES**     **All Classes** |  |


---


## **Uses of Class org.jdesktop.el.ELContext**

| Packages that use ELContext | |
| --- | --- |
| **org.jdesktop.el** | Customized version of EL for Beans Binding; not for general use. |
| **org.jdesktop.el.impl** | Customized version of EL for Beans Binding; not for general use. |
| **org.jdesktop.el.impl.lang** | Customized version of EL for Beans Binding; not for general use. |

Uses of ELContext in org.jdesktop.el

| Methods in org.jdesktop.el that return ELContext | |
| --- | --- |
| `ELContext` | `ELContextEvent.getELContext()`             Returns the `ELContext` that was created. |

| Methods in org.jdesktop.el with parameters of type ELContext | |
| --- | --- |
| `abstract  MethodExpression` | `ExpressionFactory.createMethodExpression(ELContext context, java.lang.String expression, java.lang.Class<?> expectedReturnType, java.lang.Class<?>[] expectedParamTypes)`             Parses an expression into a `MethodExpression` for later evaluation. |
| `abstract  ValueExpression` | `ExpressionFactory.createValueExpression(ELContext context, java.lang.String expression, java.lang.Class<?> expectedType)`             Parses an expression into a `ValueExpression` for later evaluation. |
| `java.lang.Class<?>` | `ResourceBundleELResolver.getCommonPropertyType(ELContext context, java.lang.Object base)`             If the base object is a ResourceBundle, returns the most general type that this resolver accepts for the `property` argument. |
| `java.lang.Class<?>` | `MapELResolver.getCommonPropertyType(ELContext context, java.lang.Object base)`             If the base object is a map, returns the most general type that this resolver accepts for the `property` argument. |
| `java.lang.Class<?>` | `ListELResolver.getCommonPropertyType(ELContext context, java.lang.Object base)`             If the base object is a list, returns the most general type that this resolver accepts for the `property` argument. |
| `abstract  java.lang.Class<?>` | `ELResolver.getCommonPropertyType(ELContext context, java.lang.Object base)`             Returns the most general type that this resolver accepts for the `property` argument, given a `base` object. |
| `java.lang.Class<?>` | `CompositeELResolver.getCommonPropertyType(ELContext context, java.lang.Object base)`             Returns the most general type that this resolver accepts for the `property` argument, given a `base` object. |
| `java.lang.Class<?>` | `BeanELResolver.getCommonPropertyType(ELContext context, java.lang.Object base)`             If the base object is not `null`, returns the most general type that this resolver accepts for the `property` argument. |
| `java.lang.Class<?>` | `ArrayELResolver.getCommonPropertyType(ELContext context, java.lang.Object base)`             If the base object is a Java language array, returns the most general type that this resolver accepts for the `property` argument. |
| `java.util.Iterator` | `ResourceBundleELResolver.getFeatureDescriptors(ELContext context, java.lang.Object base)`             If the base object is a ResourceBundle, returns an `Iterator` containing the set of keys available in the `ResourceBundle`. |
| `java.util.Iterator<java.beans.FeatureDescriptor>` | `MapELResolver.getFeatureDescriptors(ELContext context, java.lang.Object base)`             If the base object is a map, returns an `Iterator` containing the set of keys available in the `Map`. |
| `java.util.Iterator<java.beans.FeatureDescriptor>` | `ListELResolver.getFeatureDescriptors(ELContext context, java.lang.Object base)`             Always returns `null`, since there is no reason to iterate through set set of all integers. |
| `abstract  java.util.Iterator<java.beans.FeatureDescriptor>` | `ELResolver.getFeatureDescriptors(ELContext context, java.lang.Object base)`             Returns information about the set of variables or properties that can be resolved for the given `base` object. |
| `java.util.Iterator<java.beans.FeatureDescriptor>` | `CompositeELResolver.getFeatureDescriptors(ELContext context, java.lang.Object base)`             Returns information about the set of variables or properties that can be resolved for the given `base` object. |
| `java.util.Iterator<java.beans.FeatureDescriptor>` | `BeanELResolver.getFeatureDescriptors(ELContext context, java.lang.Object base)`             If the base object is not `null`, returns an `Iterator` containing the set of JavaBeans properties available on the given object. |
| `java.util.Iterator<java.beans.FeatureDescriptor>` | `ArrayELResolver.getFeatureDescriptors(ELContext context, java.lang.Object base)`             Always returns `null`, since there is no reason to iterate through set set of all integers. |
| `abstract  MethodInfo` | `MethodExpression.getMethodInfo(ELContext context)`             Evaluates the expression relative to the provided context, and returns information about the actual referenced method. |
| `Expression.Result` | `ValueExpression.getResult(ELContext context, boolean trackResolvedObjects)` |
| `abstract  java.lang.Class<?>` | `ValueExpression.getType(ELContext context)`             Evaluates the expression relative to the provided context, and returns the most general type that is acceptable for an object to be passed as the `value` parameter in a future call to the `ValueExpression.setValue(org.jdesktop.el.ELContext, java.lang.Object)` method. |
| `java.lang.Class<?>` | `ResourceBundleELResolver.getType(ELContext context, java.lang.Object base, java.lang.Object property)`             If the base object is an instance of `ResourceBundle`, return `null`, since the resolver is read only. |
| `java.lang.Class<?>` | `MapELResolver.getType(ELContext context, java.lang.Object base, java.lang.Object property)`             If the base object is a map, returns the most general acceptable type for a value in this map. |
| `java.lang.Class<?>` | `ListELResolver.getType(ELContext context, java.lang.Object base, java.lang.Object property)`             If the base object is a list, returns the most general acceptable type for a value in this list. |
| `abstract  java.lang.Class<?>` | `ELResolver.getType(ELContext context, java.lang.Object base, java.lang.Object property)`             For a given `base` and `property`, attempts to identify the most general type that is acceptable for an object to be passed as the `value` parameter in a future call to the `ELResolver.setValue(org.jdesktop.el.ELContext, java.lang.Object, java.lang.Object, java.lang.Object)` method. |
| `java.lang.Class<?>` | `CompositeELResolver.getType(ELContext context, java.lang.Object base, java.lang.Object property)`             For a given `base` and `property`, attempts to identify the most general type that is acceptable for an object to be passed as the `value` parameter in a future call to the `CompositeELResolver.setValue(org.jdesktop.el.ELContext, java.lang.Object, java.lang.Object, java.lang.Object)` method. |
| `java.lang.Class<?>` | `BeanELResolver.getType(ELContext context, java.lang.Object base, java.lang.Object property)`             If the base object is not `null`, returns the most general acceptable type that can be set on this bean property. |
| `java.lang.Class<?>` | `ArrayELResolver.getType(ELContext context, java.lang.Object base, java.lang.Object property)`             If the base object is an array, returns the most general acceptable type for a value in this array. |
| `abstract  java.lang.Object` | `ValueExpression.getValue(ELContext context)`             Evaluates the expression relative to the provided context, and returns the resulting value. |
| `java.lang.Object` | `ResourceBundleELResolver.getValue(ELContext context, java.lang.Object base, java.lang.Object property)`             If the base object is an instance of `ResourceBundle`, the provided property will first be coerced to a `String`. |
| `java.lang.Object` | `MapELResolver.getValue(ELContext context, java.lang.Object base, java.lang.Object property)`             If the base object is a map, returns the value associated with the given key, as specified by the `property` argument. |
| `java.lang.Object` | `ListELResolver.getValue(ELContext context, java.lang.Object base, java.lang.Object property)`             If the base object is a list, returns the value at the given index. |
| `abstract  java.lang.Object` | `ELResolver.getValue(ELContext context, java.lang.Object base, java.lang.Object property)`             Attempts to resolve the given `property` object on the given `base` object. |
| `java.lang.Object` | `CompositeELResolver.getValue(ELContext context, java.lang.Object base, java.lang.Object property)`             Attempts to resolve the given `property` object on the given `base` object by querying all component resolvers. |
| `java.lang.Object` | `BeanELResolver.getValue(ELContext context, java.lang.Object base, java.lang.Object property)`             If the base object is not `null`, returns the current value of the given property on this bean. |
| `java.lang.Object` | `ArrayELResolver.getValue(ELContext context, java.lang.Object base, java.lang.Object property)`             If the base object is a Java language array, returns the value at the given index. |
| `abstract  java.lang.Object` | `MethodExpression.invoke(ELContext context, java.lang.Object[] params)`             If a String literal is specified as the expression, returns the String literal coerced to the expected return type of the method signature. |
| `abstract  boolean` | `ValueExpression.isReadOnly(ELContext context)`             Evaluates the expression relative to the provided context, and returns `true` if a call to `ValueExpression.setValue(org.jdesktop.el.ELContext, java.lang.Object)` will always fail. |
| `boolean` | `ResourceBundleELResolver.isReadOnly(ELContext context, java.lang.Object base, java.lang.Object property)`             If the base object is not null and an instanceof `ResourceBundle`, return `true`. |
| `boolean` | `MapELResolver.isReadOnly(ELContext context, java.lang.Object base, java.lang.Object property)`             If the base object is a map, returns whether a call to `MapELResolver.setValue(org.jdesktop.el.ELContext, java.lang.Object, java.lang.Object, java.lang.Object)` will always fail. |
| `boolean` | `ListELResolver.isReadOnly(ELContext context, java.lang.Object base, java.lang.Object property)`             If the base object is a list, returns whether a call to `ListELResolver.setValue(org.jdesktop.el.ELContext, java.lang.Object, java.lang.Object, java.lang.Object)` will always fail. |
| `abstract  boolean` | `ELResolver.isReadOnly(ELContext context, java.lang.Object base, java.lang.Object property)`             For a given `base` and `property`, attempts to determine whether a call to `ELResolver.setValue(org.jdesktop.el.ELContext, java.lang.Object, java.lang.Object, java.lang.Object)` will always fail. |
| `boolean` | `CompositeELResolver.isReadOnly(ELContext context, java.lang.Object base, java.lang.Object property)`             For a given `base` and `property`, attempts to determine whether a call to `CompositeELResolver.setValue(org.jdesktop.el.ELContext, java.lang.Object, java.lang.Object, java.lang.Object)` will always fail. |
| `boolean` | `BeanELResolver.isReadOnly(ELContext context, java.lang.Object base, java.lang.Object property)`             If the base object is not `null`, returns whether a call to `BeanELResolver.setValue(org.jdesktop.el.ELContext, java.lang.Object, java.lang.Object, java.lang.Object)` will always fail. |
| `boolean` | `ArrayELResolver.isReadOnly(ELContext context, java.lang.Object base, java.lang.Object property)`             If the base object is a Java language array, returns whether a call to `ArrayELResolver.setValue(org.jdesktop.el.ELContext, java.lang.Object, java.lang.Object, java.lang.Object)` will always fail. |
| `abstract  void` | `ValueExpression.setValue(ELContext context, java.lang.Object value)`             Evaluates the expression relative to the provided context, and sets the result to the provided value. |
| `void` | `ResourceBundleELResolver.setValue(ELContext context, java.lang.Object base, java.lang.Object property, java.lang.Object value)`             If the base object is a ResourceBundle, throw a `PropertyNotWritableException`. |
| `void` | `MapELResolver.setValue(ELContext context, java.lang.Object base, java.lang.Object property, java.lang.Object val)`             If the base object is a map, attempts to set the value associated with the given key, as specified by the `property` argument. |
| `void` | `ListELResolver.setValue(ELContext context, java.lang.Object base, java.lang.Object property, java.lang.Object val)`             If the base object is a list, attempts to set the value at the given index with the given value. |
| `abstract  void` | `ELResolver.setValue(ELContext context, java.lang.Object base, java.lang.Object property, java.lang.Object value)`             Attempts to set the value of the given `property` object on the given `base` object. |
| `void` | `CompositeELResolver.setValue(ELContext context, java.lang.Object base, java.lang.Object property, java.lang.Object val)`             Attempts to set the value of the given `property` object on the given `base` object. |
| `void` | `BeanELResolver.setValue(ELContext context, java.lang.Object base, java.lang.Object property, java.lang.Object val)`             If the base object is not `null`, attempts to set the value of the given property on this bean. |
| `void` | `ArrayELResolver.setValue(ELContext context, java.lang.Object base, java.lang.Object property, java.lang.Object val)`             If the base object is a Java language array, attempts to set the value at the given index with the given value. |

Constructors in org.jdesktop.el with parameters of type ELContext

`ELContextEvent(ELContext source)`
  
          Constructs an ELContextEvent object to indicate that an
`ELContext` has been created.

Uses of ELContext in org.jdesktop.el.impl

| Methods in org.jdesktop.el.impl with parameters of type ELContext | |
| --- | --- |
| `MethodExpression` | `ExpressionFactoryImpl.createMethodExpression(ELContext context, java.lang.String expression, java.lang.Class expectedReturnType, java.lang.Class[] expectedParamTypes)` |
| `ValueExpression` | `ExpressionFactoryImpl.createValueExpression(ELContext context, java.lang.String expression, java.lang.Class expectedType)` |
| `MethodInfo` | `MethodExpressionLiteral.getMethodInfo(ELContext context)` |
| `MethodInfo` | `MethodExpressionImpl.getMethodInfo(ELContext context)`             Evaluates the expression relative to the provided context, and returns information about the actual referenced method. |
| `Expression.Result` | `ValueExpressionImpl.getResult(ELContext context, boolean trackResolvedObjects)` |
| `java.lang.Class` | `ValueExpressionLiteral.getType(ELContext context)` |
| `java.lang.Class` | `ValueExpressionImpl.getType(ELContext context)` |
| `java.lang.Object` | `ValueExpressionLiteral.getValue(ELContext context)` |
| `java.lang.Object` | `ValueExpressionImpl.getValue(ELContext context)` |
| `java.lang.Object` | `MethodExpressionLiteral.invoke(ELContext context, java.lang.Object[] params)` |
| `java.lang.Object` | `MethodExpressionImpl.invoke(ELContext context, java.lang.Object[] params)`             Evaluates the expression relative to the provided context, invokes the method that was found using the supplied parameters, and returns the result of the method invocation. |
| `boolean` | `ValueExpressionLiteral.isReadOnly(ELContext context)` |
| `boolean` | `ValueExpressionImpl.isReadOnly(ELContext context)` |
| `void` | `ValueExpressionLiteral.setValue(ELContext context, java.lang.Object value)` |
| `void` | `ValueExpressionImpl.setValue(ELContext context, java.lang.Object value)` |

Uses of ELContext in org.jdesktop.el.impl.lang

| Subclasses of ELContext in org.jdesktop.el.impl.lang | |
| --- | --- |
| `class` | `EvaluationContext` |

| Methods in org.jdesktop.el.impl.lang that return ELContext | |
| --- | --- |
| `ELContext` | `EvaluationContext.getELContext()` |

Constructors in org.jdesktop.el.impl.lang with parameters of type ELContext

`EvaluationContext(ELContext elContext,
FunctionMapper fnMapper,
VariableMapper varMapper,
Expression expression)`

`EvaluationContext(ELContext elContext,
FunctionMapper fnMapper,
VariableMapper varMapper,
Expression expression,
boolean trackResolvedProperties)`

`ExpressionBuilder(java.lang.String expression,
ELContext ctx)`

---


|  |  |  |
| --- | --- | --- |
| **Overview** \| **Package** \| **Class** \| **Use** \| **Tree** \| **Deprecated** \| **Index** \| **Help** | |  |
| PREV   NEXT | **FRAMES**    **NO FRAMES**     **All Classes** |  |


---
